# Preoperative moderate to severe anemia is associated with increased postoperative major adverse cardiac and cerebral events and pulmonary complications: a propensity score-matched analysis in hip fracture surgery patients over 80 years old

**DOI:** 10.1186/s13741-023-00349-5

**Published:** 2023-11-10

**Authors:** Li Min, Yang Linyi, Li Chen, Shen Jiang, Chen Chen

**Affiliations:** Department of Anesthesiology, The First People’s Hospital of Changzhou, Changzhou, Jiangsu 213003 China

**Keywords:** Anemia, Major adverse cardiac and cerebral events (MACCE), Postoperative pulmonary complications (PPCs), Hip fracture, 80 years old

## Abstract

**Background:**

Anemia is a common health problem in the elderly. Preoperative anemia is a risk factor for postoperative outcomes in the elderly for hip fracture. The objective of the study was to explore the relationship between preoperative moderate to severe anemia and postoperative morbidity and mortality in hip fracture patients over 80 years old.

**Methods:**

We performed a retrospective cohort study exploring preoperative moderate to severe anemia and postoperative morbidity and mortality. Patients over 80 years old undergoing hip fracture surgery were included in the study. Data were collected for major adverse cardiac and cerebral events (MACCE), postoperative pulmonary complications (PPCs), in-hospital mortality, delirium, gastrointestinal complication, deep venous thrombus (DVT), acute renal failure, ICU admission, and perioperative transfusion > 2 units rate.

**Results:**

A total of 912 eligible patients were included for unmatched cohort analysis, and 512 patients were included for matched cohort analysis after propensity score matching. Baseline characteristics between the normal to mild anemia and moderate to severe anemia groups were significantly different. More patients in the moderate to severe cohort had a higher ASA classification grade III and female ratio. Patients in the moderate and severe anemia cohorts had more MACCE (unadjusted: odds ratio [OR] 1.968, 96% CI 1.090–3.555,* P* 0.023; adjusted: OR 1.929, 95% CI 1.014–3.668, *P* 0.045) and PPCs (unadjusted: OR 2.616, 95% CI 1.442–4.748, *P* 0.001; adjusted: OR 2.352, 95% CI 1.225–4.516, *P* 0.010) than patients with normal or mild anemia. However, the transfusion > 2 units rate was not significantly different between the two cohorts (unadjusted: OR 0.967, 95% CI 0.737–1.270, *P* 0.811; adjusted: OR 0.941, 95% CI 0.693–1.278, *P* 0.697). The in-hospital mortality, delirium rate, gastrointestinal complication, ICU admission, and DVT were similar. However, the in-hospital mortality was much higher (3.6%, 21/591 vs 1.6%, 5/321) in the moderate to severe anemia cohort. Furthermore, after propensity score-matched analysis, MACCE and PPCs were also significantly increased in the moderate to severe anemia cohort (OR 2.196 & 3.171, 95% CI 1.0794.470 & 1.563–6.436, *P* 0.027 & 0.001), which were in accordance with the unadjusted and adjusted results in the unmatched cohorts.

**Conclusions:**

Moderate to severe preoperative anemia (< 11 g/dl) is associated with increased postoperative major adverse cardiac and cerebral events and pulmonary complications. Additionally, in-hospital mortality was not significant but was higher in the preoperative moderate to severe anemia cohort. Preoperative assessment and correction of hemoglobin level to above 11 g/dl might reduce MACCE, PPCs, and in-hospital mortality in hip fracture patients over 80 years old.

## Introduction

The prevalence of anemia in surgery patients is relatively high, especially in elderly patients (Nguyen et al. 2021; Bailey et al. 2021; Sincavage et al. 2021). The anemic prevalence could be more than 50% in aged hip fracture patients, as previously reported (Sim et al. 2018). Preoperative anemia is associated with poor clinical outcomes in cardiac and non-cardiac surgery patients (Ljungqvist et al. 2021). Hip fracture is a major injury in elderly patients over 80 years old, and the morbidity and mortality rates are relatively high in such patients (Menéndez-Colino et al. 2021; Veronese and Maggi 2018). Preoperative anemia is associated with an increased likelihood of adverse outcomes, and it increases with the severity of anemia (Schmerler et al. 2023). The association of preoperative anemia and an increased risk of in-hospital mortality, myocardial infarction, stroke, and infection was found in a meta-analysis of patients undergoing major surgery (Fowler et al. 2015).

Elderly hip fracture patients are commonly in a poor preoperative physical state resulting from trauma, blood loss, pain, relevant complications, and comorbidities. Patients over 80 years old are a frail group with cardiopulmonary, mental, vascular, psychological, and cognitive diseases. Preoperative anemia is a modifiable factor that can be improved in the preoperative period in hip fracture patients.

Timing for hip fracture surgery is recommended by guidelines within 48 h (Griffiths et al. 2021). Optimizing the anemic condition of preoperative patients may be beneficial for hip fracture patients over 80 years old, but postponing surgery may cause adverse events. The preoperative management program has been complemented by our osteoarthropathy center since 2016. The aims of the research were to (1) evaluate the effect of preoperative anemia on the postoperative major adverse cardiac and cerebral events (MACCE) and postoperative pulmonary complications (PPCs), (2) assess whether the delay of the surgery in hip fracture patients over 80 years old is associated with higher in-hospital mortality, and (3) explore the relationship between preoperative anaemia and postoperative delirium rate, ICU admission rate, gastrointestinal complication, deep venous thrombus (DVT) rate, and transfusion rate > 2 units.

## Methods

### Design and setting

This was a single-center retrospective cohort study conducted in an academic tertiary center. The study was performed in accordance with the Strengthening the Reporting of Observational Studies in Epidemiology (STROBE) Statement of cohort studies. The study protocol was reviewed and approved by the ethics committee of the hospital with a waiver of informed consent.

### Study population and eligibility criteria

After approval from the ethics board of the tertiary teaching hospital and informed consent from patients, data were collected from the database of the clinical electronic system for the observational study. Two trained senior doctors independently accessed the database and acquired the data. Controversial issues were discussed according to the predefined protocol. Further entry into the patients’ health system was conducted if more information on the comorbidities and medical history were needed. Telephone interviews were followed for details if necessary. Consecutive surgical hip fracture patients over 80 years old from 2016 to 2020 were recruited. The inclusion criteria were as follows: (1) hip fracture patients receiving surgery and (2) age over 80 years old. The exclusion criteria were as follows: (1) refusal to participate in the research, (2) canceling of the surgery, (3) multiple injuries, (4) receiving surgery within 3 months, (5) receiving other surgeries during the admission period, and (6) revision arthroplasty or second unilateral surgery.

### Definition

Anaemia is defined as a hemoglobin level of less than 12 g/dl for women and less than 13 g/dl in men by the World Health Organization (Shander et al. 2011). A preoperative hemoglobin level of less than 11 g/dl is suggested as moderate to severe anemia for both sexes. Mild anemia of hemoglobin levels between 11 to 12 g/dl for women and 12 to 13 g/dl for men were not included in the analysis for the purpose of the study, sex-neutral and age-relevant consideration. Lung function was assessed preoperatively with a respiratory meter. The results of the patients were classified as having normal, mild decline, moderate decline, or severe decline in lung function. Hip fracture was diagnosed with ICD-10, and the surgery type included internal fixation, hemiarthroplasty, and arthroplasty. Anesthesia was implemented with general anesthesia (with or without nerve blocks) or epidural anesthesia alone.

The primary outcomes of the study were major adverse cardiac and cerebral events (MACCE) and postoperative pulmonary complications (PPCs). Secondary outcomes included the relationship between preoperative anemia and postoperative delirium rate, ICU admission rate, gastrointestinal complication, and deep venous thrombus (DVT) rate. MACCE was reported as a composite of heart failure, myocardial infarction, new onset of arrhythmias affecting hemodynamics, stroke, or death from any cause (Shander et al. 2011). PPCs were defined with clinical manifestation and imaging examination as pneumonia, hemothorax, hydrothorax, pulmonary atelectasis, acute respiratory distress syndromes, and respiratory failure requiring ventilation. Delirium was diagnosed with DSM-V [Diagnostic and Statistical Samuel of Mental Disorders fifth edition].

### Statistical analysis

Continuous variables were expressed as the means (SD) or medians (IQR) for normally and non-normally distributed variables, respectively. Percentages were used to describe category variables. Continuous variables between cohorts were compared with the *t* test or Mann‒Whitney *U* test, and binary variables were compared with the chi-square test or Fisher’s exact test as appropriate. Factors with *P* < 0.05 in the univariate analysis were entered as covariables into the multivariable logistic regression for the endpoints. The in-hospital mortality between the unmatched cohorts was compared using Kaplan‒Meier curves. To assess the consistency of the results, a propensity score-matched analysis was conducted. A calliper of 0.02 was determined to limit the differences between the matched pairs. Variables that were included for the scoring were similar to those for the multivariable models. All analyses were conducted with SPSS version 26.0. The significance level was defined as a *P* value < 0.05.

## Results

### Unmatched cohorts

**Fig. 1:**
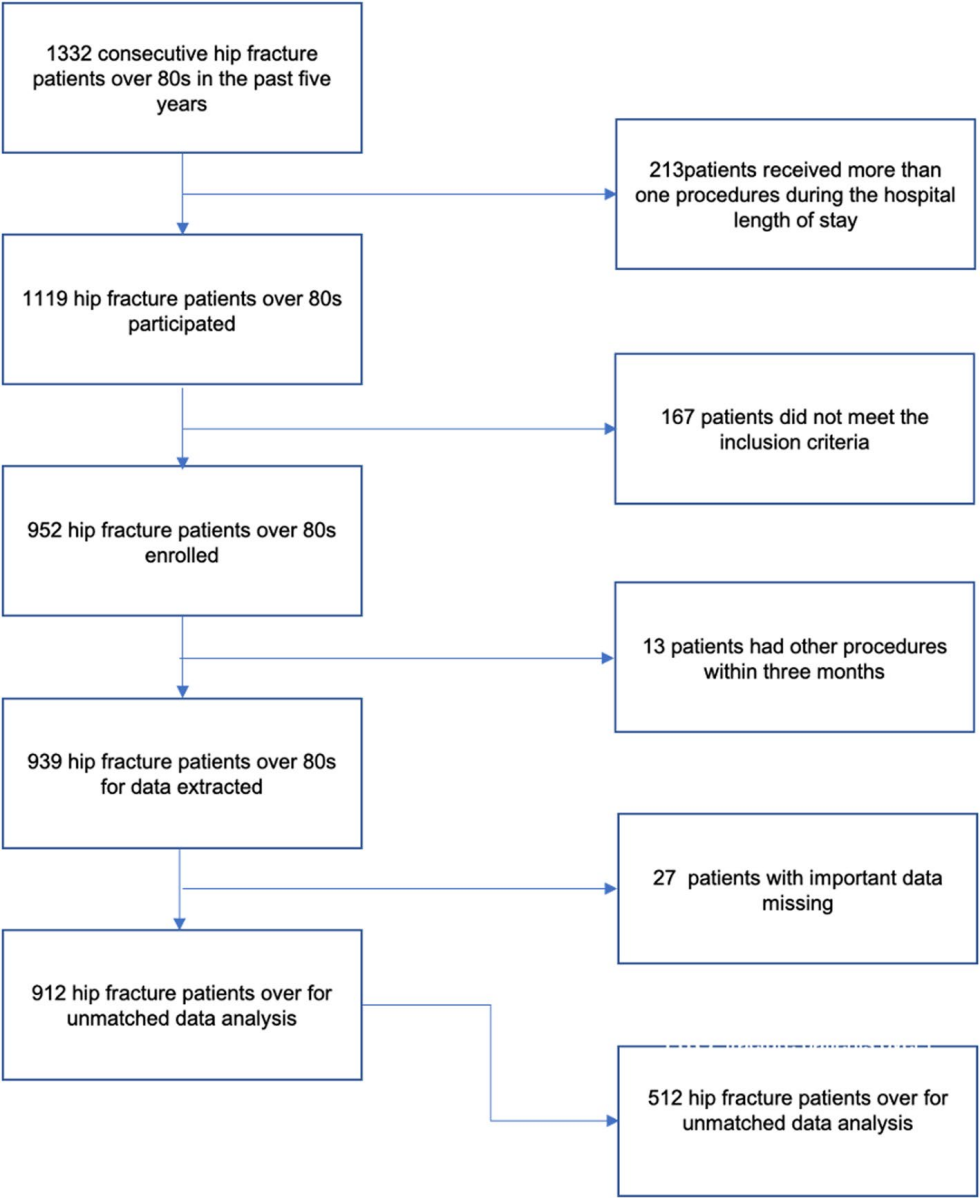
Flow chart of included hip fracture patients over 80 years old

From 2016 to 2020, 912 eligible patients were included for unmatched cohort analysis, and 512 patients were included for matched cohort analysis after propensity score matching. A total of 420 patients were excluded if they had missing baseline data or met the exclusion criteria. The detailed enrollment flowchart is shown in Fig. 1. Baseline characteristics and relevant surgery record data are shown in Table 1. The mean (SD) age of the included hip fracture patients was 85.35 (3.91) years old, the median (IQR) hemoglobin level was 106 (92,120) g/dl, and the percentage of moderate to severe anemia with a preoperative hemoglobin level < 11 g/dl was 64.8% (591/912). Baseline characteristics between the normal to mild anemia and moderate to severe anemia groups were significantly different. More patients in the moderate to severe cohort had a higher ASA classification grade III and female ratio. However, the normal to mild anemia cohort had longer preoperative days, which might have resulted from preoperative blood management. In addition, more chronic obstructive pulmonary disease (COPD), hypertension, heart failure, atrial fibrillation, stroke, chronic kidney failure, and decreased lung function were observed in the moderate to severe anemia cohort. It is likely that intertrochanteric fracture causes moderate to severe anemia. Patients in the moderate to severe anemic cohort intended to have more epidural anesthesia and nerve blocks (Table 1).

**Table 1:** Baseline characteristics of hip fracture patients over 80 years old in unmatched cohorts

	Hemoglobin (g/dl)		*P* value
	< 11 (*n* = 591)	≥ 11 (*n* = 321)	
Age, years	85.64 (3.9)	84.86 (3.7)	0.002
ASA physical status			
II	292 (49.4)	184 (57.3)	0.031
III	298 (50.4)	136 (42.4)	
IV	1 (0.2)	1 (0.3)	
Female sex	459 (77.7)	210 (65.4)	< 0.001
Preoperative days	4 [2,5]	4 [3,5]	0.004
COPD	156 (26.4)	105 (32.7)	0.044
Hypertension	318 (53.8)	194 (60.4)	0.054
Diabetes	98 (16.6)	71 (22.1)	0.040
CAD	46 (7.8)	25 (7.8)	0.998
Heart failure	6 (1.0)	2 (0.6)	0.814
Atrial fibrillation	16 (2.7)	3 (0.9)	0.073
Stroke	89 (15.1)	42 (13.1)	0.417
Chronic kidney failure	8 (1.4)	0 (0.0)	0.085
Cancer	27 (4.6)	16 (5.0)	0.777
Dementia	16 (2.7)	9 (2.8)	0.932
Depression	2 (0.3)	2 (0.6)	0.616
Parkinson	12 (2.0)	8 (2.5)	0.649
Pneumonia	12 (2.0)	6 (1.9)	0.867
Lung function			
No to mild lung function decline	417 (70.6)	201 (62.6)	0.013
Moderate lung function decline	88 (14.9)	72 (22.4)	
Severe lung function decline	86 (14.6)	48 (15.0)	
Diagnosis			
Femoral neck fracture	184 (31.1)	216 (67.3)	< 0.001
Intertrochanteric fracture	386 (65.3)	101 (31.5)	
Subtrochanteric fracture	2 (0.3)	1 (0.3)	
Other	19 (3.2)	3 (0.9)	
Anesthesia technique			
With nerve block	321 (54.3)	136 (42.4)	< 0.001
Without nerve block	157 (26.6)	128 (39.9)	
Epidural Anesthesia	113 (19.1)	57 (7.8)	
Surgery type			
Internal fixation	402 (68.0)	110 (34.3)	< 0.001
Hemiarthroplasty	155 (26.2)	165(51.4)	
Arthroplasty	34 (5.8)	46 (14.3)	
Duration of anesthesia, min	160 [125,195]	170 [135,205]	0.001
Duration of surgery, min	50 [40,65]	55 [40,65]	0.004

*Abbreviations: COPD* Chronic obstructive pulmonary disease, *CAD* Coronary artery disease

**Fig. 2:**
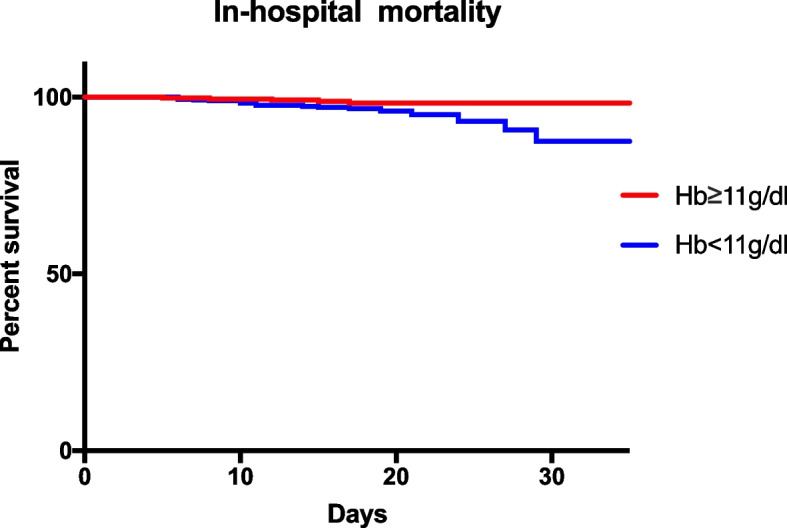
In-hospital mortality of hip fracture patients over 80 years old

According to unadjusted results and adjusted results before matching, patients in the moderate and severe anemia cohorts had more MACCE (unadjusted: OR 1.968, 96% CI 1.090–3.555,* P* 0.023; adjusted: OR 1.929, 95% CI 1.014–3.668, *P* 0.045) and PPCs (unadjusted: OR 2.616, 95% CI 1.442–4.748, *P* 0.001; adjusted: OR 2.352, 95% CI 1.225–4.516, *P* 0.010) than patients with normal or mild anemia. Although the normal or mild anemia patients received more transfusions in the preoperative period, the total transfusion > 2 U rate was not significantly different between the two cohorts (unadjusted: OR 0.967, 95% CI 0.737–1.270, *P* 0.811; adjusted: OR 0.941, 95% CI 0.693–1.278, *P* 0.697). The in-hospital mortality, delirium rate, gastrointestinal complication, ICU admission, and DVT were similar (Table 2). However, the in-hospital mortality was higher (3.6%, 21/591 vs 1.6%, 5/321) in the moderate to severe anemia cohort than in the normal to mild anemia cohort (Fig. 2).

**Table 2:** Postoperative outcomes in unmatched cohorts

Dichotomous outcomes	Unadjusted results		*P* value	Adjusted results		*P* value
	Odds ratio	95%CI		Odd ratio	95%CI	
MACCE	1.968	1.090–3.555	0.023	1.929	1.014–3.668	0.045
PPCs	2.616	1.442–4.748	0.001	2.352	1.225–4.516	0.010
In-hospital Mortality	2.328	0.870–6.235	0.084	2.651	0.908–7.736	0.074
Delirium	0.935	0.276–3.270	1.000	0.952	0.241–3.766	0.945
GI complication	0.950	0.276–3.270	1.000	0.662	0.159–2.766	0.572
ICU	1.639	0.441–6.098	0.660	3.894	0.783–19.359	0.097
DVT	2.010	0.557–7.259	0.421	1.730	0.421–7.111	0.447
Transfusion (> 2U)	0.967	0.737–1.270	0.811	0.941	0.693–1.278	0.697

*Abbreviations: MACCE* Major adverse cardiac and cerebral events, *PPCs* Postoperative pulmonary complications, *GI* Gastrointestinal, *DVT* Deep venous thrombus

### Propensity score-matched analysis

After propensity score matching, 512 patients with normal to mild anemia and moderate to severe anemia were enrolled in the matched cohorts. The baseline characteristics of hip fracture patients over 80 years old were balanced between the normal/mild anemia cohort and the moderate to severe anemia cohort (Table 3). In the unmatched cohort, patients in the normal to severe anemia cohort were older, had a higher ASA grade, were more likely to be female, and had more COPD, hypertension, heart failure, atrial fibrillation, stroke, chronic kidney failure, pneumonia, decreased lung function, intertrochanteric fracture, nerve blocks, epidural anesthesia, and internal fixation surgery.

**Table 3:** Baseline characteristics of hip fracture patients over 80 years old in matched cohorts

	Hemoglobin (g/dl)		*P* value
	< 11 (*n* = 265)	≥ 11 (*n* = 265)	
Age, years	84.9 (3.7)	85.1 (3.7)	0.251
ASA physical status			
II	153 (57.7)	148 (55.8)	0.661
III	112 (42.3)	117 (44.2)	
Female sex	78 (29.4)	74 (27.9)	0.701
Preoperative days	4 [3,5]	4 [3,5]	0.972
COPD	84 (31.7)	75 (28.3)	0.394
Hypertension	150 (56.6)	157 (59.2)	0.538
Diabetes	48 (18.1)	51 (19.2)	0.738
CAD	17 (6.4)	18 (6.8)	0.861
Heart failure	3 (1.1)	2 (0.8)	> 0.999
Atrial fibrillation	3 (1.1)	3 (1.1)	> 0.999
Stroke	41 (15.5)	34 (12.8)	0.383
Cancer	16 (6.0)	15 (5.7)	0.853
Dementia	5 (1.9)	6 (2.3)	0.761
Depression	0 (0)	2 (0.8)	0.499
Parkinson	7 (2.6)	8 (3.0)	0.793
Pneumonia	6 (2.3)	3 (1.1)	0.504
Lung function			
No to mild lung function decline	177 (66.8)	169 (63.8)	0.173
Moderate lung function decline	42 (15.8)	58 (21.9)	
Severe lung function decline	46 (17.4)	38 (14.3)	
Diagnosis			
Femoral neck fracture	148 (55.8)	161 (60.8)	0.645
Intertrochanteric fracture	114 (43.0)	100 (37.7)	
Subtrochanteric fracture	1 (0.4)	1 (0.4)	
Other	2 (0.8)	3 (1.1)	
Anesthesia technique			
With nerve block	123 (46.4)	112 (42.3)	0.355
Without nerve block	90 (34.0)	106 (40.0)	
Epidural anesthesia	52 (19.6)	47 (17.7)	
Surgery type			
Internal fixation	116 (43.8)	109 (41.4)	0.612
Hemiarthroplasty	118 (44.5)	129 (48.7)	
Arthroplasty	31 (11.7)	27 (10.2)	
Duration of anesthesia, min	165 [136,207]	165 [135,200]	0.980
Duration of surgery, min	55 [40,65]	55 [40,65]	0.559

*Abbreviations: COPD* Chronic obstructive pulmonary disease, *CAD* Coronary artery disease

MACCE and PPCs were significantly increased in the moderate to severe anemia cohort (OR 2.196 & 3.171, 95% CI 1.079–4.470 & 1.563–6.436, *P* 0.027 & 0.001), which was in accordance with the unadjusted and adjusted results in the unmatched cohorts. However, the in-hospital mortality rate was higher (2 vs 10) in the moderate to higher cohort in the matched cohorts. The controversial conclusion might result from the relatively small sample size. The other complications, such as delirium, gastrointestinal complication, ICU admission, DVT, and transfusion > 2 U, were in accordance with the results in the unmatched cohorts.

## Discussion

In our observational study, we found that preoperative moderate to severe anemia in hip fracture surgical patients over 80 years old was associated with more MACCE and PPCs. In addition, preoperative moderate to severe anemic patients had higher in-hospital mortality than those with normal to mild anemia. However, according to the study, the patients had similar rates of delirium, ICU admission, gastrointestinal complications, and DVT between cohorts.

The prevalence of preoperative anemia in our study was much higher than reported. One reason was that the target population in our study was hip fracture patients, and another reason was that the research focused on patients over 80 years old. The concept of anemia is defined by the WHO as a hemoglobin level less than 12 g/dl in women and 13 g/dl in men. It was recommended by the blood management in hip fracture patients the preoperative hemoglobin level should be kept above 8 g/dl, and 10 g/dl was suggested for frail patients with serious comorbidities. However, the threshold for blood management in hip fracture patients over 80 years old is unknown. A higher hemoglobin level of 11 g/dl was cautiously chosen for hip fracture patients over 80 years old because it is a clinically implicated level that could compromise both men and women. It was hypothesized that anemia-induced tissue hypoxia may contribute to the pathophysiology associated with clinical anemia (Hare et al. 2018). Acute kidney injury, stroke, and myocardial injury may occur because of anemia (Hare Gregory 2021).

Perioperative anemia has long been an issue that captures both surgeons’ and anaesthethiologists’ attention. Several original studies and reviews have reported increased postoperative morbidity and mortality in both cardiac and noncardiac surgery patients with abnormal preoperative hemoglobin concentrations. Padmanabhan and colleagues observed anemic impacts on morbidity and mortality after surgery with less improvement, and preoperative correction of anemia could improve outcomes of cardiac surgery (Padmanabhan et al. 2016). Moreover, Burton and colleagues suggested that preoperative anemia might be associated with a higher comorbidity burden and the likelihood of postoperative morbidity and mortality (Burton et al. 2019). Another study by Fowler and colleagues included 38,770 patients from 474 hospitals in 27 countries, and 30% were anemic. They found an increased risk of complications and death (Fowler et al. 2018).

Anemia was associated with vital organ injury. Musallam and colleagues (Musallam et al. 2011) collected data from a prospective registry from 211 hospitals for a 30-day mortality and morbidity. This was a retrospective cohort study with 227,425 patients and 30.4% preoperative anemia compared with 64.7% preoperative anemia of hemoglobin < 11 g/dl in our study. In agreement with the study, we also reported higher in-hospital mortality in anemic patients than in patients over 80 years old with preoperative Hb concentrations greater than 11 g/dl. Both studies were retrospective studies, but their research was a multicenter study with more patients. Noncardiac surgery was included, but our study focused on hip fracture surgery, with more elderly patients over 80 years old. Multivariate logistic regression was used to assess the adjusted effect of anemia, but our study was conducted with a propensity score-matched analysis to confirm the strength of the results. They also found that preoperative anemia was associated with increased postoperative morbidity and mortality, as in our study. Patients over 80 years old accounted for less than 3% in the study, and orthopedics surgery accounted for less than 5% of the surgeries. As pointed out by the authors, 7% of the preoperative hematocrit concentrations were obtained more than 4 weeks before surgery and might not accurately predict the concentrations at the time of surgery. However, in our study of hip fracture surgery and in elderly patients over 80 years old, all hemoglobin values were obtained within 3 days before surgery and were more strongly associated with patient outcomes. Sameed and colleagues (Sameed et al. 2021) reported that postoperative pulmonary complications contributed substantially to perioperative morbidity, mortality, and health care costs. Compared to advanced age, physical condition, specific comorbidities, and surgery type, preoperative anemia when treated decreases the risk of adverse outcomes in surgical patients. It was reported that in noncardiac surgery, the prevalence of anemia might be over 30% with an increased prevalence in elderly patients, which was over 65% in our study. Carson and colleagues (Boyd-Carson et al. 2020) reported preoperative anemia (hemoglobin < 11 g/dl) with morbidity and mortality after emergency surgery and concluded that anemia was associated with increased a 90-day and a 30-day mortality, prolonged hospital stay, and risk of return to the operating theater.

It is paradoxical that transfusion and anemia are associated with organ injury and increased morbidity and mortality in surgical interventions (Shander et al. 2011). The transfusion rate (> 2 U) was similar in both cohorts, but the timeline of the treatment was not analyzed in the study. It was reported that preoperative blood management with acute therapy near the time of surgery decreased the intraoperative transfusion rate and increased the postoperative hemoglobin level (Hare Gregory 2021). Several studies have focused on the relationship of postoperative transfusion and mobility and mortality with different conclusions. Smeets and colleagues found no effect of erythrocyte blood transfusion on mortality for hip fracture (Smeets et al. 2018). Bolliger and colleagues suggested a beneficial effect of perioperative transfusion for gastrectomy for cancer (Kouyoumdjian et al. 2021). However, Dukleska and colleagues reported increased morbidity and mortality with preoperative blood transfusions in neonates undergoing surgery (Dukleska et al. 2020). Although timely diagnosis and preoperative anemia treatment are necessary for surgical patients, implementation of anemia therapy is challenging partly because of limited evidence (Bolliger et al. 2022) (Table 4).

**Table 4:** Postoperative complications in matched cohorts

	Hemoglobin (g dl^−1^)		Odd ratio	95%CI	*P* value
	< 11 (*n* = 256)	≥ 11 (*n* = 256)			
MACCE	25 (9.4)	12 (4.5)	2.196	1.079–4.470	0.027
PPCs	32 (12.1)	11 (4.2)	3.171	1.563–6.436	0.001
In-hospital mortality	10 (3.8)	2 (0.8)	5.157	1.119–23.766	0.019
Delirium	3 (1.1)	3 (1.1)	0.991	0.249–3.940	> 0.999
GI complication	5 (1.9)	4 (1.5)	1.255	0.333–4.725	> 0.999
ICU	7 (2.6)	1 (0.4)	7.163	0.875–58.672	0.068
DVT	5 (1.9)	2 (0.8)	2.529	0.486–13.151	0.450
Transfusion (> 2U)	123 (46.4)	124 (46.8)	1.015	0.722–1.428	0.931

*Abbreviations: MACCE* Major adverse cardiac and cerebral events, *PPCs* Postoperative pulmonary complications, *GI* Gastrointestinal, *DVT* Deep venous thrombus

This study has several limitations. First, it is a retrospective single-center study; thus, the cause and effect relationship cannot be confirmed in our study. Second, the time span of the study period was approximately 5 years. The clinical practice guidelines were updated, and the threshold of the transfusion was revised by year, especially for cardiac, orthopedic, and critically ill patients. However, transfusion was also analyzed in our practice, and multiple statistical analyses were used to lessen the effect of the unknown confounders. In addition, all the known factors that affected the outcomes of hip fractures were included.

## Conclusions

In conclusion, we report that a lower preoperative hemoglobin concentration (< 11 g/dl) is associated with increased postoperative major adverse cardiac and cerebral events and pulmonary complications. Additionally, in-hospital mortality was not significant but was higher in the lower hemoglobin cohort. As a modifiable component, preoperative assessment and correction of hemoglobin levels to normal values might reduce MACCE, PPCs, and in-hospital mortality in hip fracture patients over 80 years old.

## Abbreviations

MACCE: Major adverse cardiac and cerebral events
PPCs: Postoperative pulmonary complications
DVT: Deep venous thrombus
STROBE: Strengthening the Reporting of Observational Studies in Epidemiology
COPD: Chronic obstructive pulmonary disease
ASA: American Society of Anesthesiologists
CAD: Coronary artery disease
GI: Gastrointestinal
